# Thermal Aggregation of Hen Egg White Proteins in the Presence of Salts

**DOI:** 10.1007/s10930-015-9612-3

**Published:** 2015-05-22

**Authors:** Kazuki Iwashita, Naoto Inoue, Akihiro Handa, Kentaro Shiraki

**Affiliations:** Faculty of Pure and Applied Sciences, University of Tsukuba, 1-1-1 Tennodai, Tsukuba, Ibaraki 305-8573 Japan; R&D Division, Institute of Technology, Kewpie Corporation, 2-5-7 Sengawa, Chofu, Tokyo 182-0002 Japan

**Keywords:** Hen egg white proteins, Thermal aggregation, Hofmeister series, High concentration, Crowding

## Abstract

Hen egg white contains more than 40 kinds of proteins with concentrations reaching 100 mg/mL. Highly concentrated protein mixtures are common in the food industry, but the effects of a crowded environment containing salts on protein stability and aggregation have only been investigated using pure protein solutions. Here, we investigated the thermal aggregation of hen egg white protein (EWP) at various concentrations in the presence of inorganic salts by solubility measurements and SDS-PAGE. EWP at 1 mg/mL formed aggregates with increasing temperature above 55 °C; the aggregation temperatures increased in the presence of inorganic salt with the Hofmeister series. Namely, the chaotrope 0.5 M NaSCN completely suppressed the thermal aggregation of 1 mg/mL EWP. As the protein concentration increased, NaSCN unexpectedly enhanced the protein aggregation; the aggregation temperature of 10 and 100 mg/mL EWP solutions were dramatically decreased at 62 and 47 °C, respectively. This decrease in aggregation temperatures due to the chaotrope was described by the excluded volume effect, based on a comparative experiment using Ficoll 70 as a neutral crowder. By contrast, the kosmotrope Na_2_SO_4_ did not affect the aggregation temperature at concentrations from 1 to 100 mg/mL EWPs. The unexpected fact that a chaotrope rather enhanced the protein aggregation at high concentration provides new insight into the aggregation phenomena with the Hofmeister effect as well as the crude state of highly concentrated proteins.

## Introduction

Physiologic fluids in living systems are so crowded with biomacromolecules that a significant fraction of the intracellular space is not available to other macromolecules [1–3]. This crowded environment is known to greatly affect protein stability and aggregation by altering the kinetics and the equilibrium parameters compared to dilute solution [4, 5]. The effect of a crowded environment on protein stability has mainly been described from an excluded volume perspective, in which a solution contains only one kind of protein with a high concentration of inert macromolecules, typically polyethyleneglycol, dextran, or Ficoll as crowders to mimic crowded conditions [6, 7]. However, the environment in living systems is, in fact, highly crowded with the heterogeneous macromolecules, i.e., proteins, nucleic acids, lipids, and polysaccharides [8]. This heterogeneous and highly concentrated condition is also relevant to the food industry [9]. Protein stability and aggregation in this crude environment will be investigated by protein biophysics to understand the intrinsic behaviors in living organisms.

The crowded environment affects protein stability and aggregation by specific interactions between cosolutes and proteins, as well as the excluded volume effect. The specific effect of cosolutes on protein stability has been classically described by Yancey as osmolytic [10]. Such small-molecular-weight cosolutes have been extensively investigated with regard to protein aggregation, such as amino acids [11, 12] and their derivatives [13], arginine [14–17] and its derivatives [18, 19], and amine compounds [20–22]. These data revealed that small organic additives decrease the aggregation rate of protein and increase the solubility of the aggregation-prone unfolded protein. The design rule of aggregation suppressors remained obscure: (1) multivalent amines decrease the aggregation rate to suppress chemical modification; (2) the amino acid backbone is an indispensable structure as an additive for heat-induced aggregation; and (3) the guanidine group increases the solubility of aromatic compounds. However, inorganic salts have comparatively simple rules in terms of their effect on protein aggregation. For example, the surface tension of various kinds of saline describes the thermal aggregation of egg white lysozyme [23].

The Hofmeister series is a well-known index for additive effects on protein aggregation; the propensity of aggregation is as follows [24, 25]:

$${\text{CO}}_{3}^{2 - } > {\text{SO}}_{4}^{2 - } > {\text{S}}_{2} {\text{O}}_{3}^{2 - } > {\text{H}}_{2} {\text{PO}}_{4}^{ - } > {\text{F}}^{ - } > {\text{Cl}}^{ - } > {\text{Br}}^{ - } \approx {\text{NO}}_{3}^{ - } > {\text{I}}^{ - } > {\text{ClO}}_{4}^{ - } > {\text{SCN}}^{ - }$$

In general, chaotropes (typically SCN^−^ and I^−^) show the so-called “salting-in” effect that destabilizes protein tertiary structure, leading to a decrease in the denaturation temperature. In contrast, kosmotropes (typically CO_3_^2−^ and SO_4_^2−^) show a “salting-out” effect that stabilizes protein structure [26]. Hofmeister series have been applied in various protein industries and research fields for purification and stabilization in fundamental research, such as the strength of ionic hydration [27, 28], different density of water molecules [29, 30], and accumulation or exclusion of ions from the surface [31, 32].

In this study, we have investigated the thermal aggregation of high and low concentrations of hen egg white proteins with several types of inorganic salts. The egg white proteins were chosen because of the existing protein systems [33]. Egg white from the domestic chicken is one of the most prominent protein source foods. The biophysical structure of egg white plays an important role in the functional properties of food, such as water-holding, emulsifying, foaming, and gelation due to high protein concentration (100 mg/mL) [34–36]. In addition, egg white contains various kinds of proteins with various molecular weights, isoelectric points, and concentrations [9, 37–39]. Ovalbumin, with a molecular weight of 45 kDa, is the most abundant protein, accounting for half the content of egg white proteins. Ovotransferrin and ovomucoid are the next most abundant proteins, with molecular weights of 76 and 28 kDa, respectively. Small amounts of dozens other proteins have been identified, although egg white has the favorable property of no lipid content. This type of crude condition with heterogeneous proteins is common in daily life. Several papers have been reported about the egg white proteins, such as thermal aggregation of egg white proteins [40] and NMR structure of a model protein in the presence of egg white as crowding agent [41]. By contrast, this study provides the first attempt to understand the biophysical aspects of the aggregation highly concentrated protein with Hofmeister salts. This study provides the first attempt to understand the biophysical aspects of highly concentrated protein aggregation with Hofmeister salts.

## Materials and Methods

### Materials

Sodium thiocyanate (NaSCN), sodium chloride (NaCl), sodium sulfate (Na_2_SO_4_), and magnesium chloride (MgCl_2_) were obtained from Wako Pure Chemical Industries Ltd. (Osaka Japan). 2-[4-(2-Hydroxyethyl)-1-piperazinyl]ethanesulfonic acid (HEPES) was obtained from Nacalai Tesque (Kyoto, Japan). Ficoll 70 with an average molecular weight of 70 kDa and egg white ovalbumin (grade III) were obtained from Sigma Chemical Co. (St. Louis, MO).

### Preparation of Hen Egg White Proteins

Hen egg white proteins were prepared by the following procedure to obtain samples for reproducible experiments. Hen egg white protein (EWP) was diluted with an equal volume of distilled water, stirred gently with a magnetic stirrer for 1 h at 4 °C, and then dialyzed using a 1000 MW cut-off dialyzed tube against distilled water with four changes at 4 °C to remove small-molecular-weight compounds and salts. The samples were then centrifuged at 10,000×*g* for 30 min to remove undesirable large aggregates for the spectroscopic analysis of the following experiments. It is noted that the protein contents of EWP after the centrifugation is almost identical to that of pristine sample. The supernatant was freeze-dried and then used for further experiments.

### Thermal Aggregation of Hen Egg White Proteins

The freeze-dried EWP was dissolved in 0.5 M sodium salts (NaSCN, NaCl, and Na_2_SO_4_), 10 mM MgCl_2_, and 20 mM HEPES and adjusted to the appropriate protein concentration at pH 7.4. The small amount of divalent ion (MgCl_2_) was added in all conditions due to the understanding of the structural change of protein under physiological condition. Samples in the presence of 150 mg/mL Ficoll 70 containing 1 mg/mL EWP with 0.5 M sodium salts, 10 mM MgCl_2_, and 20 mM HEPES were also prepared and adjusted to pH 7.4. Aliquots of 80 μL of the solutions were added to microfuge tubes. The EWP solutions were heated at various temperatures for 30 min using a temperature control system (GeneAtlasG; Astec, Fukuoka, Japan). After the heat treatment, the samples were stirred with a spatula and centrifuged at 15,000×*g* for 20 min at 25 °C. The supernatant concentration of proteins was then analyzed by measuring the absorbance at 280 nm (A_280_) using a spectrophotometer (ND-1000, NanoDrop Technologies, Inc., Wilmington, Del, USA). The relative absorbance values (A/A_0_ × 100) were plotted in the figures; A and A_0_ show the absorbance of the sample in the presence of salt after and before the heat treatment, respectively.

### Sodium Dodecyl Sulfate–Polyacrylamide Gel Electrophoresis

The supernatants of the protein solutions after the heat treatment were dissolved in 62.5 mM Tris–HCl (pH 6.8) loading buffer containing 2 % (w/v) SDS, 5 % sucrose, 5 % β-mercaptoethanol, and 0.01 % bromophenol blue. The samples were heated for 5 min in boiling water and then subjected to sodium dodecyl sulfate polyacrylamide gel electrophoresis (SDS-PAGE) using a 5–20 % gradient gel (e-PAGEL, ATTO Co., Tokyo, Japan) with a molecular weight marker (Precision Plus Protein Dual Xtra Standards; BIO-RAD, Hercules, CA, USA). The gels were then stained using silver nitrate.

## Results

### Thermal Aggregation of Egg White Proteins

**Fig. 1:**
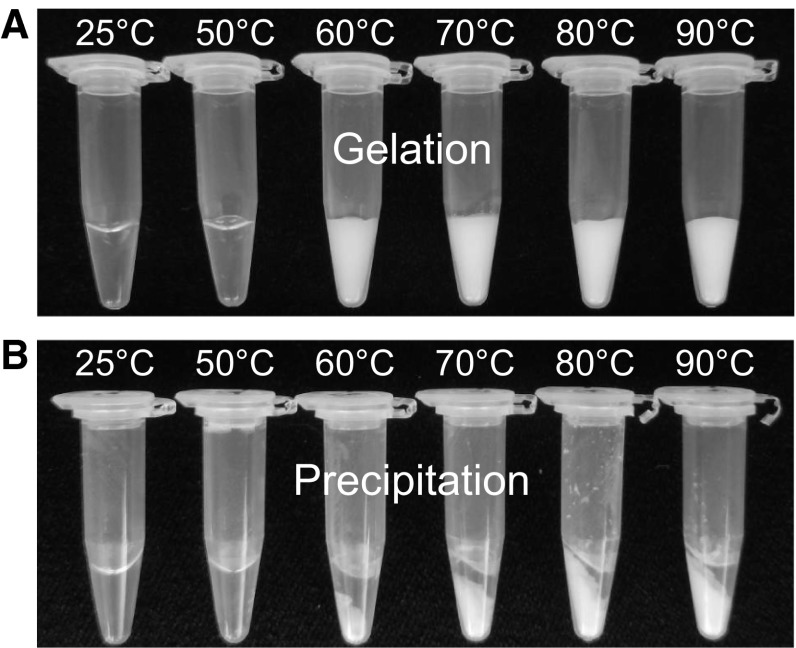
Gelation of the EWP solution at 100 mg/mL in the absence of sodium salts. **a** After heat treatment for 30 min. **b** After centrifugation at 15,000×*g* for 20 min

We investigated the concentration-dependent thermal aggregation of EWP in the presence of the inorganic salts NaSCN, NaCl, and Na_2_SO_4_. It is noted that NaSCN and Na_2_SO_4_ are chaotrope and kosmotrope, respectively, with the propensities of salting-in and salting-out at the high salt concentration of 0.5 M. As shown in Fig. 1, high-concentration EWP was easily gelled by heat treatment. The samples of 100 mg/mL EWPs without salts were visually similar, with white turbidity, after the heat treatment at 60–90 °C (Fig. 1a). The centrifuged samples were separated from the protein pellet with a clear supernatant in the absence of salts (Fig. 1b). It should be noted that MgCl_2_ slightly accelerates the protein aggregation by the heat treatment.

**Fig. 2:**
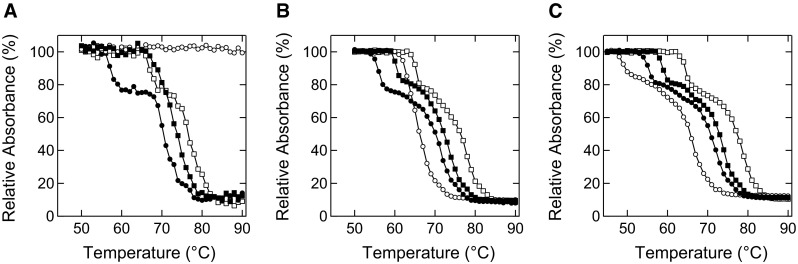
Supernatant absorbance of EWP after heat treatment. The samples containing 0.5 M NaSCN (*open circles*), NaCl (*closed squares*), Na_2_SO_4_ (*open squares*), and no additive (*closed circles*) were heat treated for 30 min at the respective temperatures. **a** 1 mg/mL. **b** 10 mg/mL. **c** 100 mg/mL

Figure 2 shows the concentration of soluble proteins after heat treatment for 30 min at the respective temperatures. EWP without salt additive began to aggregate at approximately 55 °C at all protein concentrations examined (1, 10, and 100 mg/mL). These data were unexpected because highly concentrated proteins are also prone to form aggregates due to the increased probability of protein–protein interaction. However, this independence of protein concentration implies the possibility that the rate-limiting step of aggregation is an unfolding reaction rather than protein–protein interaction, similar to the reaction-limited cluster–cluster aggregation of protein [42].

The addition of 0.5 M salts to 1 mg/mL EWP led to a change in aggregation temperature (Fig. 2a). As expected, NaSCN completely inhibited the thermal aggregation of 1 mg/mL EWP even at 90 °C. NaCl and Na_2_SO_4_ resulted in the aggregation of 1 mg/mL EWP above 65 °C, and the absorbance decreased to 10 % at 90 °C. The starting point temperatures of aggregation increased in the order Na_2_SO_4_ ~ NaCl < NaSCN, which corresponds to the sequence of these salts in the Hofmeister series.

The aggregation tendency of 10 mg/mL EWP with the addition of salt (Fig. 2b) was different from the aggregation tendency of 1 mg/mL EWP, especially with NaSCN. EWP at 10 mg/mL with NaSCN showed marked aggregation at 62 °C, whereas 1 mg/mL EWP with NaSCN did not aggregate even at 90 °C. The aggregation temperature of 10 mg/mL EWP with NaCl was decreased by 8 °C compared to 1 mg/mL EWP with NaCl. However, the results for 10 mg/mL EWP with Na_2_SO_4_ were almost identical to the results for 1 mg/mL EWP with Na_2_SO_4_.

The aggregation curves of 100 mg/mL EWP without salt and with NaCl and Na_2_SO_4_ (Fig. 2c) were similar to the aggregation curves of 10 mg/mL EWP with each salt (Fig. 2b). However, the aggregation temperature of 100 mg/mL EWP with NaSCN decreased compared to the aggregation temperature for 10 mg/mL EWP with NaSCN. Interestingly, the start point temperatures of aggregation increased in the order NaSCN < NaCl < Na_2_SO_4_, which was the inverse sequence of the Hofmeister series.

The data shown in Fig. 2 can be summarized as follows. (1) EWP without salt showed the same aggregation curves regardless of protein concentration from 1 to 100 mg/mL. (2) EWP with Na_2_SO_4_ showed similar aggregation curves regardless of protein concentration from 1 to 100 mg/mL. However, EWP with NaSCN showed a decrease in aggregation temperature depending on EWP concentration. (3) The order of aggregation temperatures was Na_2_SO_4_ < NaCl < NaSCN at the low concentration (1 mg/mL) of EWP. (4) In contrast, the order of aggregation temperatures was NaSCN < NaCl < Na_2_SO_4_ at the high concentration (100 mg/mL) of EWP.

### SDS-PAGE of Egg White Proteins

**Fig. 3:**
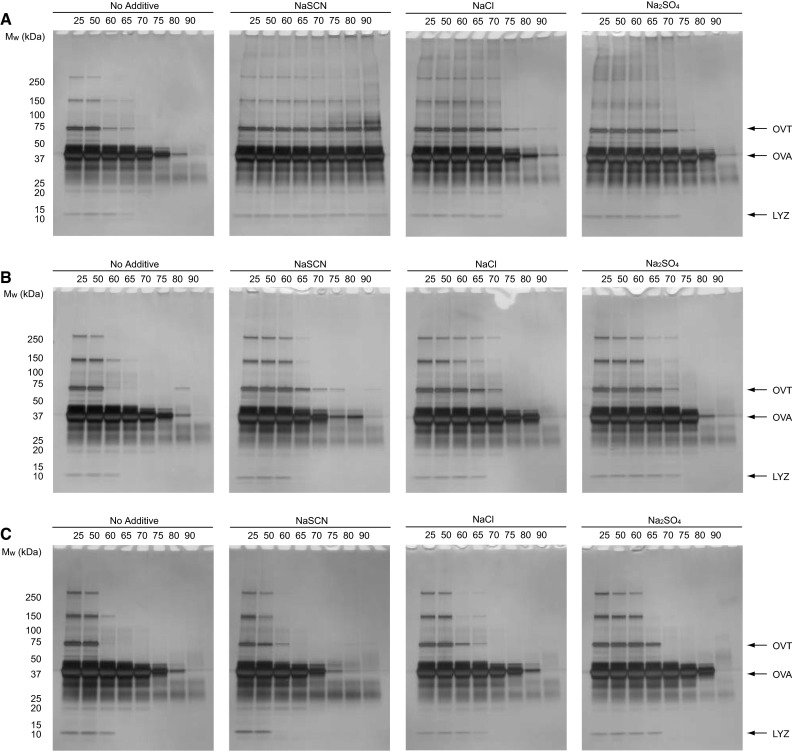
SDS-PAGE analyses of heat-induced aggregation of EWP with 0.5 M sodium salts. **a** 1 mg/mL. **b** 10 mg/mL. **c** 100 mg/mL. The *numbers* in the figures show the temperature of heat treatment (°C). *OVT*, *OVA*, and *LYZ* indicate ovotransferrin, ovalbumin, and lysozyme, respectively

Table 1 shows the properties of the major proteins in EWP, ovalbumin (OVA), ovotransferrin (OVT), ovomucoid, and lysozyme (LYZ). To determine the aggregation propensities of the individual proteins, we performed SDS-PAGE analysis of the EWP after heat treatment. Samples containing 1, 10, and 100 mg/mL EWP in 500 mM salts were prepared and subjected to heat treatment for 30 min at different temperatures; the samples were then centrifuged and the supernatant analyzed by SDS-PAGE (Fig. 3). The bands of OVA, OVT, and LYZ were successfully separated at approximately 45, 76, and 14 kDa, respectively. The SDS-PAGE patterns of NaSCN samples at 1 mg/mL EWP did not change even at 90 °C for 30 min, while the bands of OVA and OVT in the NaCl and Na_2_SO_4_ samples decreased with increasing temperature of the heat treatment. To more clearly see this behavior, the aggregation temperatures of OVA, OVT, and LYZ in 1 mg/mL EWP appeared to be in the order Na_2_SO_4_ ~ NaCl < NaSCN (Table 2). EWP at 10 mg/mL sample in the presence of NaSCN decreased the all bands, which was similar pattern to the presence of NaCl and Na_2_SO_4_. Further increasing concentration of EWP (100 mg/mL) in the presence of NaSCN decreased the overall bands comparing to Na_2_SO_4_ and NaCl. The aggregation temperatures of OVA, OVT, and LYZ in 10 and 100 mg/mL EWP were in the order NaSCN < NaCl < Na_2_SO_4_ (Table 2). These data can be summarized as follows: the low concentration of EWP was aggregated by the kosmotrope, while the high concentration of EWP was aggregated by the chaotrope, regardless of the kind of protein in EWP.

**Table 1:** Major proteins of EWP

Protein	% of egg white protein^a^	Isoelectric point^a,b,c^	Molecular weight (kDa)^a,b,c^	Denaturation temperature (°C)^a^
Ovalbumin	54.0	4.5 (5.19)	45.0 (42.9)	84.0
Ovotransferrin	12.0	6.1 (6.85)	76.0 (77.8)	61.0
Ovomucoid	11.0	4.1 [4.82]	28.0 [20.0]	79.0
Lysozyme	3.4	10.7	14.3	75.0

^a^Data are from Rao [55]

^b^Data shown in parentheses are from Ning Qiu [56]

^c^Data shown in square brackets are from Catherine Guérin-Dubiard [9]

**Table 2:** Apparent order of the thermal aggregation propenscity analysed by SDS-PAGE

Protein	1 mg/mL	10 mg/mL	100 mg/mL
OVT	Na_2_SO_4_ ~ NaCl < NaSCN	NaSCN ~ NaCl < Na_2_SO_4_	NaSCN < NaCl < Na_2_SO_4_
OVA	Na_2_SO_4_ ~ NaCl < NaSCN	NaSCN < NaCl < Na_2_SO_4_	NaSCN < NaCl < Na_2_SO_4_
LYZ	Na_2_SO_4_ ~ NaCl < NaSCN	NaSCN < NaCl < Na_2_SO_4_	NaSCN < NaCl < Na_2_SO_4_

### Excluded Volume Effect of Ficoll 70

**Fig. 4:**
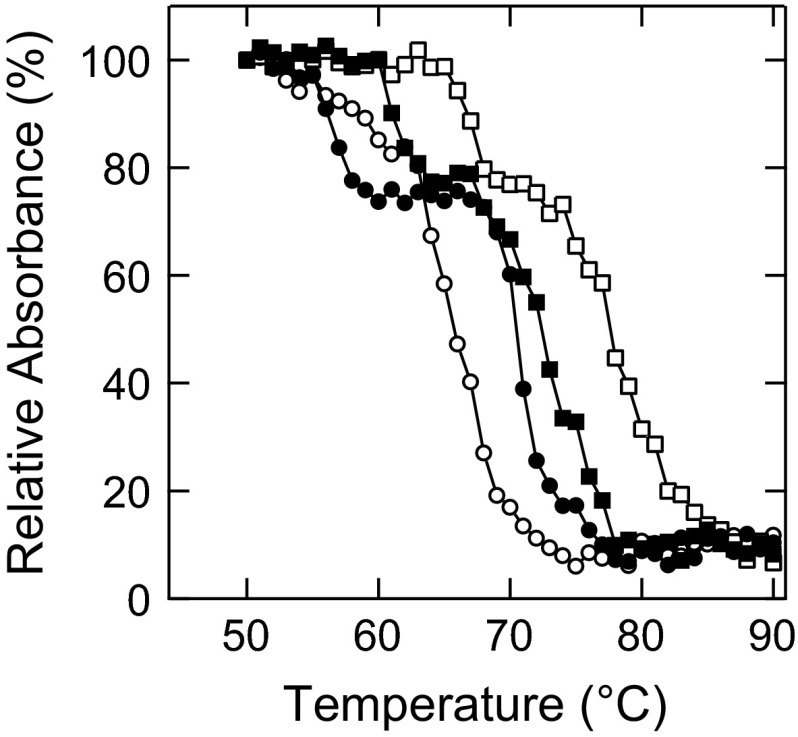
Supernatant absorbance of 1 mg/mL EWP with 150 mg/mL Ficoll 70 after heat treatment. The samples containing 0.5 M NaSCN (*open circles*), NaCl (*closed squares*), Na_2_SO_4_ (*open squares*), and no additive (*closed circles*) were heat treated for 30 min at the respective temperatures

The concentration-dependent behavior of the thermal aggregation of EWP by salts was investigated from the perspective of the excluded volume effect. Ficoll 70 has been used as a hydrophilic polysaccharide for its excluded volume effect with the non-specific steric repulsion of protein molecules [43, 44]. Samples containing 1 mg/mL EWP with 150 mg/mL Ficoll 70 in the presence or absence of salts were prepared and heated for 30 min. Figure 4 shows the supernatant concentration of centrifuged protein solutions after the heat treatment. The aggregation curve of 1 mg/mL EWP with 150 mg/mL Ficoll 70 without salt (Fig. 4) was similar to 1 mg/mL EWP without Ficoll 70 and salt (Fig. 2a). By contrast, the aggregation curve of 1 mg/mL EWP with 150 mg/mL Ficoll 70 with NaSCN (Fig. 4) was different from 1 mg/mL EWP without Ficoll 70 and salt (Fig. 2a). Accordingly, Ficoll 70 promoted EWP aggregation in the presence of NaSCN. In the presence of NaCl, Ficoll 70 slightly enhanced the aggregation of EWP compared to 1 mg/mL EWP (Figs. 2a, 4). Interestingly, Ficoll 70 did not affect the thermal aggregation profiles of EWP in the presence of Na_2_SO_4_ (Figs. 2a, 4). Thus, the excluded volume effect enhanced aggregation only in the presence of NaSCN.

**Fig. 5:**
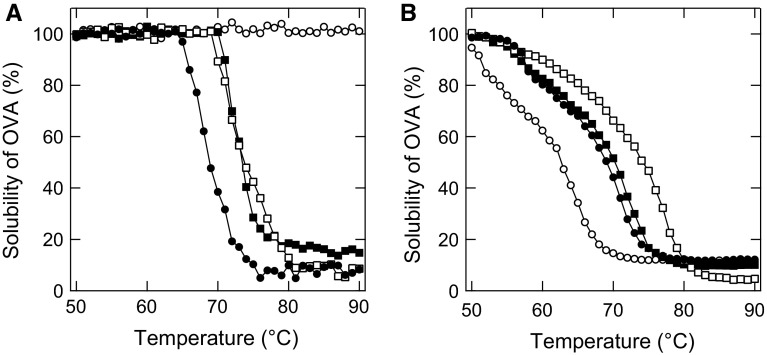
Solubility of OVA after heat treatment. The samples containing 0.5 M NaSCN (*open circles*), NaCl (*closed squares*), Na_2_SO_4_ (*open squares*), and no additive (*closed circles*) were heat treated for 30 min at the respective temperatures. **a** 1 mg/mL. **b** 100 mg/mL

To clarify the crowding effect, we investigated the thermal aggregation of purified OVA alone. Figure 5 shows the supernatant concentrations of 1 and 100 mg/mL OVA after heat treatment for 30 min at different temperatures. The sample of OVA without salt aggregated at 60–70 °C. In the presence of NaSCN, 1 mg/mL OVA did not aggregate at 90 °C (Fig. 5a), which is a similar pattern to the EWP shown in Fig. 2a. In contrast, 100 mg/mL OVA was prone to form aggregates (Fig. 5b), which was different from the patterns in the presence of NaCl and Na_2_SO_4_. These data support the hypothesis that chaotropes actually promote protein aggregation by the excluded volume effect.

## Discussion

This study was performed to investigate the thermal aggregation of crude EWP at 100 mg/mL in comparison to 1 mg/mL in the presence of different types of salts. The results can be summarized as follows. (1) The order of the Hofmeister series on thermal aggregation was altered by protein concentration. (2) The chaotrope NaSCN was unexpectedly the most aggregation-prone additive for the high protein concentration. (3) The inverse Hofmeister effect was attributed to the macromolecular excluded volume effect.

The most interesting finding of this study is the data regarding the effects of NaSCN on the thermal aggregation of EWP. NaSCN suppressed thermal aggregation at a low concentration of EWP, but not a high concentration (Fig. 2). This concentration-dependent aggregation of protein can be discussed in terms of cluster-aggregation theory as follows. Thermal aggregation generally occurs with the initial formation of start aggregates with a diameter of approximately 50 nm at the initial stage, followed by the growth of the aggregates to large size through association of the start aggregates [42]. This growth of aggregates is classified into diffusion-limited cluster aggregation (DLCA) and reaction-limited cluster aggregation (RLCA); the rate-limiting step of DLCA is the encounter rate of start aggregates, and the rate-limiting step of RLCA is the association and reaction rate of start aggregates [45, 46]. In this situation, it is naturally thought that the crowded environment decreases the diffusion rate, while at the same time, the crowded environment increases the probability of protein–protein interaction [2, 47–50]. Thus, it may be that a chaotrope increases the RLCA-type aggregation in the crowded environment.

Finally, it should be noted that the Hofmeister inverse series has been reported for the cloud-point temperature of lysozyme [51–53]. At a high concentration of salts above 0.5 M, a kosmotrope increases the surface tension of the solution compared to a chaotrope [54], leading to salting-out of protein molecules, which is the direct Hofmeister series effect. At a low concentration of salts, anions bind to the positively-charged surface of lysozyme, leading to increased solubility regardless of the type of ions, which is the inverse Hofmeister series effect. These data show the electrostatic interaction between protein and ions, which affects the solubility of the protein. However, our data describe protein–protein interaction with thermally-unfolded proteins at high ion concentrations. At a high protein concentration, small aggregates are prone to form further aggregates in the presence of a chaotrope, as discussed above. This inverse Hofmeister effect described by RLCA-type aggregation will be found for various proteins at high concentration, as similar data were obtained for both the crude mixture of EWP (Fig. 2) and a model crowded environment with albumin and Ficoll 70 (Figs. 4, 5).

The thermal aggregation of protein is an important phenomenon in the food industry. However, the control of aggregation is difficult even for pure protein in typical biophysical studies. We believe that this study provides important information on aggregation in high-concentration protein mixtures. Similar data are not expected for biophysical experiments at diluted concentrations of pure protein in vitro. In conclusion, the crowded environment unexpectedly increased the probability of protein aggregation in the presence of a chaotrope. The thermal aggregation of highly concentrated protein mixtures is a notable issue in both the food industry and the intercellular environment.

## Abbreviations

EWP: Egg white protein
OVA: Ovalbumin
OVT: Ovotransferrin
LYZ: Lysozyme

## References

[1] Ellis RJ (2001). Macromolecular crowding: obvious but underappreciated. Trends Biochem Sci.

[2] Minton AP (2001). The influence of macromolecular crowding and macromolecular confinement on biochemical reactions in physiological media. J Biol Chem.

[3] Rivas G, Ferrone F, Herzfeld J (2004). Life in a crowded world. EMBO Rep.

[4] Chebotareva NA, Kurganov BI, Livanova NB (2004). Biochemical effects of molecular crowding. Biochemistry.

[5] Breydo L, Reddy KD, Piai A, Felli IC, Pierattelli R, Uversky VN (2014). The crowd you’re in with: effects of different types of crowding agents on protein aggregation. Biochim Biophys Acta.

[6] Perham M, Stagg L, Wittung-Stafshede P (2007). Macromolecular crowding increases structural content of folded proteins. FEBS Lett.

[7] Homouz D, Perham M, Samiotakis A, Cheung MS, Wittung-Stafshede P (2008). Crowded, cell-like environment induces shape changes in aspherical protein. Proc Natl Acad Sci USA.

[8] Ellis RJ, Minton AP (2003). Cell biology: join the crowd. Nature.

[9] Guérin-Dubiard C, Pasco M, Mollé D, Désert C, Croguennec T, Nau F (2006). Proteomic analysis of hen egg white. J Agric Food Chem.

[10] Yancey PH, Clark ME, Hand SC, Bowlus RD, Somero GN (1982). Living with water stress: evolution of osmolyte systems. Science.

[11] Shiraki K, Kudou M, Fujiwara S, Imanaka T, Takagi M (2002). Biophysical effect of amino acids on the prevention of protein aggregation. J Biochem.

[12] Ito L, Shiraki K, Yamaguchi H (2010). Comparative analysis of amino acids and amino-acid derivatives in protein crystallization. Acta Crystallogr Sect F Struct Biol Cryst Commun.

[13] Matsuoka T, Hamada H, Matsumoto K, Shiraki K (2009). Indispensable structure of solution additives to prevent inactivation of lysozyme for heating and refolding. Biotechnol Prog.

[14] Hirano A, Arakawa T, Shiraki K (2008). Arginine increases the solubility of coumarin: comparison with salting-in and salting-out additives. J Biochem.

[15] Ariki R, Hirano A, Arakawa T, Shiraki K (2011). Arginine increases the solubility of alkyl gallates through interaction with the aromatic ring. J Biochem.

[16] Tomita S, Nagasaki Y, Shiraki K (2012). Different mechanisms of action of poly(ethylene glycol) and arginine on thermal inactivation of lysozyme and ribonuclease A. Biotechnol Bioeng.

[17] Arakawa T, Kita Y (2014). Multi-faceted arginine: mechanism of the effects of arginine on protein. Curr Protein Pept Sci.

[18] Shiraki K, Kudou M, Nishikori S, Kitagawa H, Imanaka T, Takagi M (2004). Arginine ethylester prevents thermal inactivation and aggregation of lysozyme. Eur J Biochem.

[19] Hamada H, Shiraki K (2007). L-argininamide improves the refolding more effectively than l-arginine. J Biotechnol.

[20] Kudou M, Shiraki K, Fujiwara S, Imanaka T, Takagi M (2003). Prevention of thermal inactivation and aggregation of lysozyme by polyamines. Eur J Biochem.

[21] Okanojo M, Shiraki K, Kudou M, Nishikori S, Takagi M (2005). Diamines prevent thermal aggregation and inactivation of lysozyme. J Biosci Bioeng.

[22] Hamada H, Takahashi R, Noguchi T, Shiraki K (2008). Differences in the effects of solution additives on heat- and refolding-induced aggregation. Biotechnol Prog.

[23] Hirano A, Hamada H, Okubo T, Noguchi T, Higashibata H, Shiraki K (2007). Correlation between thermal aggregation and stability of lysozyme with salts described by molar surface tension increment: an exceptional propensity of ammonium salts as aggregation suppressor. Protein J.

[24] Kunz W, Henle JWNB, Ninham BW (2004). “Zur lehre von der wirkung der salze” (about the science of the effect of salts): Franz Hofmeister’s historical papers. Curr Opin Colloid Interface Sci.

[25] Lo Nostro P, Ninham BW (2012). Hofmeister phenomena: an update on ion specificity in biology. Chem Rev.

[26] Jungwirth P, Cremer PS (2014). Beyond hofmeister. Nat Chem.

[27] Kunz W (2010). Specific ion effects in colloidal and biological systems. Curr Opin Colloid Interface Sci.

[28] Schwierz N, Horinek D, Netz RR (2013). Anionic and cationic hofmeister effects on hydrophobic and hydrophilic surfaces. Langmuir.

[29] Zhang Y, Cremer PS (2006). Interactions between macromolecules and ions: the Hofmeister series. Curr Opin Chem Biol.

[30] López-León T, Santander-Ortega MJ, Ortega-Vinuesa JL, Bastos-González D (2008). Hofmeister effects in colloidal systems: influence of the surface nature. J Phys Chem C.

[31] Pegram LM, Record MT (2008). Quantifying accumulation or exclusion of H^+^, HO^−^, and Hofmeister salt ions near interfaces. Chem Phys Lett.

[32] Nihonyanagi S, Yamaguchi S, Tahara T (2014). Counterion effect on interfacial water at charged interfaces and its relevance to the hofmeister series. J Am Chem Soc.

[33] Abeyrathne EDNS, Lee HY, Ahn DU (2013). Egg white proteins and their potential use in food processing or as nutraceutical and pharmaceutical agents–a review. Poultry Sci.

[34] Handa A, Takahashi K, Kuroda N, Froning GW (1998). Heat-induced egg white gels as affected by pH. J Food Sci.

[35] Croguennec T, Nau F, Brule G (2002). Influence of pH and salts on egg white gelation. Food Eng Phys Prop.

[36] Sun Y, Hayakawa S (2002). Heat-induced gels of egg white/ovalbumins from five avian species: thermal aggregation, molecular forces involved, and rheological properties. J Agric Food Chem.

[37] Mann K (2007). The chicken egg white proteome. Proteomics.

[38] Qiu N, Ma M, Zhao L, Liu W, Li Y, Mine Y (2012). Comparative proteomic analysis of egg white proteins under various storage temperatures. J Agric Food Chem.

[39] Wang J, Wu J (2014). Proteomic analysis of fertilized egg white during early incubation. EuPA Open Proteomics.

[40] Mine Y, Noutomi T, Haga N (1990). Thermally induced changes in egg white proteins. J Agric Food Chem.

[41] Sanfelice D, Adrover M, Martorell G, Pastore A, Temussi PA (2012). Crowding versus molecular seeding: NMR studies of protein aggregation in hen egg white. J Phys Condens Matter.

[42] Tomita S, Yoshikawa H, Shiraki K (2011). Arginine controls heat-induced cluster-cluster aggregation of lysozyme at around the isoelectric point. Biopolymers.

[43] van den Berg B, Ellis RJ, Dobson CM (1991). Effects of macromolecular crowding on protein folding and aggregation. EMBO J.

[44] Sarkar M, Li C, Pielak GJ (2013). Soft interactions and crowding. Biophys Rev.

[45] Lin MY, Lindsay HM, Weitz DA, Ball RC, Klein R, Meakin P (1989). Universality in colloid aggregation. Nature.

[46] Markossian KA, Yudin IK, Kurganov BI (2009). Mechanism of suppression of protein aggregation by α-crystallin. Int J Mol Sci.

[47] Ellis RJ (2001). Macromolecular crowding: an important but neglected aspect of the intracellular environment. Curr Opin Struct Biol.

[48] Kozer N, Schreiber G (2004). Effect of crowding on protein–protein association rates: fundamental differences between low and high mass crowding agents. J Mol Biol.

[49] Wang Y, Li C, Pielak GJ (2010). Effects of proteins on protein diffusion. J Am Chem Soc.

[50] Wang Y, Benton LA, Singh V, Pielak GJ (2012). Disordered protein diffusion under crowded conditions. J Phys Chem Lett.

[51] Zhang Y, Cremer PS (2009). The inverse and direct Hofmeister series for lysozyme. Proc Natl Acad Sci.

[52] Schwierz N, Horinek D, Netz RR (2010). Reversed anionic Hofmeister series: the interplay of surface charge and surface polarity. Langmuir.

[53] Boström M, Parsons DF, Salis A, Ninham BW, Monduzzi M (2011). Possible origin of the inverse and direct Hofmeister series for lysozyme at low and high salt concentrations. Langmuir.

[54] Pegram LM, Record MT (2007). Hofmeister salt effects on surface tension arise from partitioning of anions and cations between bulk water and the air–water interface. J Phys Chem B.

[55] Rao Q, Rocca-Smith JR, Labuza TP (2012). Moisture-induced quality changes of hen egg white proteins in a protein/water model system. J Agric Food Chem.

[56] Qiu N, Ma M, Cai Z, Jin Y, Huang X, Huang Q, Sun S (2012). Proteomic analysis of egg white proteins during the early phase of embryonic development. J Proteomics.

